# Subjective Cognitive Impairment in Liver Transplant Recipients

**DOI:** 10.1093/geroni/igaa057.516

**Published:** 2020-12-16

**Authors:** Dami Ko, Mary Dietrich, Katherine Gifford, Sheila Ridner

**Affiliations:** 1 Northeastern University, Jamaica Plain, Massachusetts, United States; 2 Vanderbilt University, Nashville, Tennessee, United States

## Abstract

Cognitive impairment is an emerging health concern in the liver transplant (LT) population. To successfully address this condition and improve patient-centered outcomes (e.g., quality of life), it is essential to involve LT recipients and caregivers in developing care plans for cognitive impairment and take into account their subjective ratings regarding recipients’ cognitive function. Although a high degree of agreement between recipient-rated and caregiver-rated cognitive function is desirable to reduce conflicts in developing plans, disagreement between the two may exist. This cross-sectional study is the first to examine the correlation between recipient-rated and caregiver-rated cognitive function in the LT population. Sixty pairs of adult LT recipients (mean age 60.4±6.9) and their caregivers (mean relationship with recipients 35 years) participated in this study. Two versions of Modified Everyday Cognition (ECog), one for LT recipients and the other for caregivers, were used to assess recipient-rated and caregiver-rated cognitive function in six domains, including memory and planning. Significant intra-class correlations were found in the ECog total and domain scores. The correlation coefficient of the ECog total score was 0.48, indicating a fair correlation, and the coefficients of the ECog domains ranged between 0.35 and 0.56, indicating poor to fair correlations based on the guideline of Cicchetti (1994). These findings suggest that a tailored approach to addressing the poor agreement between recipients and caregivers should be adopted to develop successful treatment plans for cognitive impairment. Future studies should examine the degree of agreement between objective (e.g., cognitive tests) and subjective ratings of cognitive function.

